# Drugs targeting CTGF in the treatment of pulmonary fibrosis

**DOI:** 10.1111/jcmm.18448

**Published:** 2024-05-22

**Authors:** Yudan Qiu, Yueyue Que, Zheyu Ding, Shanshan Zhang, Rong Wei, Jianing Xia, Yingying Lin

**Affiliations:** ^1^ School of Pharmacy Hangzhou Normal University Hangzhou Zhejiang China; ^2^ Key Laboratory of Elemene Class Anti‐Cancer Chinese Medicines; Engineering Laboratory of Development and Application of Traditional Chinese Medicines; Collaborative Innovation Center of Traditional Chinese Medicines of Zhejiang Province Hangzhou Normal University Hangzhou Zhejiang China

**Keywords:** CTGF, drugs, pulmonary fibrosis, treatment

## Abstract

Pulmonary fibrosis represents the final alteration seen in a wide variety of lung disorders characterized by increased fibroblast activity and the accumulation of substantial amounts of extracellular matrix, along with inflammatory damage and the breakdown of tissue architecture. This condition is marked by a significant mortality rate and a lack of effective treatments. The depositing of an excessive quantity of extracellular matrix protein follows the damage to lung capillaries and alveolar epithelial cells, leading to pulmonary fibrosis and irreversible damage to lung function. It has been proposed that the connective tissue growth factor (CTGF) plays a critical role in the advancement of pulmonary fibrosis by enhancing the accumulation of the extracellular matrix and exacerbating fibrosis. In this context, the significance of CTGF in pulmonary fibrosis is examined, and a summary of the development of drugs targeting CTGF for the treatment of pulmonary fibrosis is provided.

## 1 INTRODUCTION

Pulmonary fibrosis includes primary pulmonary fibrosis, secondary fibrosis, idiopathic fibrosis, interstitial pulmonary fibrosis and interstitial pneumonia. Idiopathic pulmonary fibrosis (IPF), a chronic, progressive fibrotic disorder of the interstitial lung, results in patients having an average lifespan of 3–5 years.^1^ Although numerous causes of pulmonary fibrosis have been identified through ongoing research, the development of effective medications remains limited. Nintedanib and pirfenidone are the main medications currently utilized in clinical settings. The disease's progression involves complex mechanisms and pathways, including disrupted epithelial repair, impaired host defence, cellular senescence and imbalanced immune responses, such as the activation of macrophage subsets and fibroproliferative response kinases associated with abnormalities.^2^ The activation of the transforming growth factor (TGF) and its subsequent pro‐fibrotic and developmental pathways contribute to the development of pulmonary fibrosis.^3^

Connective tissue growth factor (CTGF) exerts a broad impact on cell migration, adhesion and proliferation, establishing it as a critical element in the development of fibrotic diseases such as renal fibrosis,^4^ cardiac fibrosis,^5^ and pulmonary fibrosis, among others. There exist numerous reviews on CTGF's involvement in renal and cardiac fibrosis. However, its precise function in pulmonary fibrosis remains less clearly defined.

Transforming growth factor‐β (TGF‐β) and CTGF, both recognized as profibrotic growth factors, function downstream of the nuclear translocation of β‐catenin, leading to enhanced fibrogenesis.^6^ CTGF, a downstream effector of TGF‐β, serves as a matricellular protein that influences the function of growth factors, adhesion molecules, integrins, and the extracellular matrix (ECM); thus, playing a pivotal role in tissue remodelling and fibrosis.^7^ In models of pulmonary fibrosis, CTGF is often utilized to demonstrate the severity of the condition. Studies and reports on drugs targeting CTGF have also been conducted.^8^ Elucidating CTGF's role in pulmonary fibrosis is deemed crucial for the exploration of drugs targeting CTGF for the treatment of this condition.

### 1.1 Pulmonary fibrosis

Pulmonary fibrosis represents the end‐stage of lung diseases characterized by the proliferation of fibroblasts and the accumulation of substantial ECM, alongside inflammatory damage and the destruction of tissue architecture.^9^ In essence, when healthy alveolar tissue is damaged and heals improperly, structural abnormalities arise, leading to respiratory failure and, potentially, death. It has been reported that the COVID‐19 virus may induce and exacerbate pulmonary fibrosis, increasing the risk of mortality.^10^ IPF, as a form of pulmonary fibrosis, is associated with several complications, including pulmonary hypertension, chronic obstructive pulmonary disease (COPD) and lung cancer.^11^ Currently, Nintedanib and Pirfenidone are prescribed to slow the decline in lung function, reduce mortality and lower the risk of acute exacerbation (AE).^12^

### 1.2 The molecular mechanism underlying pulmonary fibrosis

**FIGURE 1:**
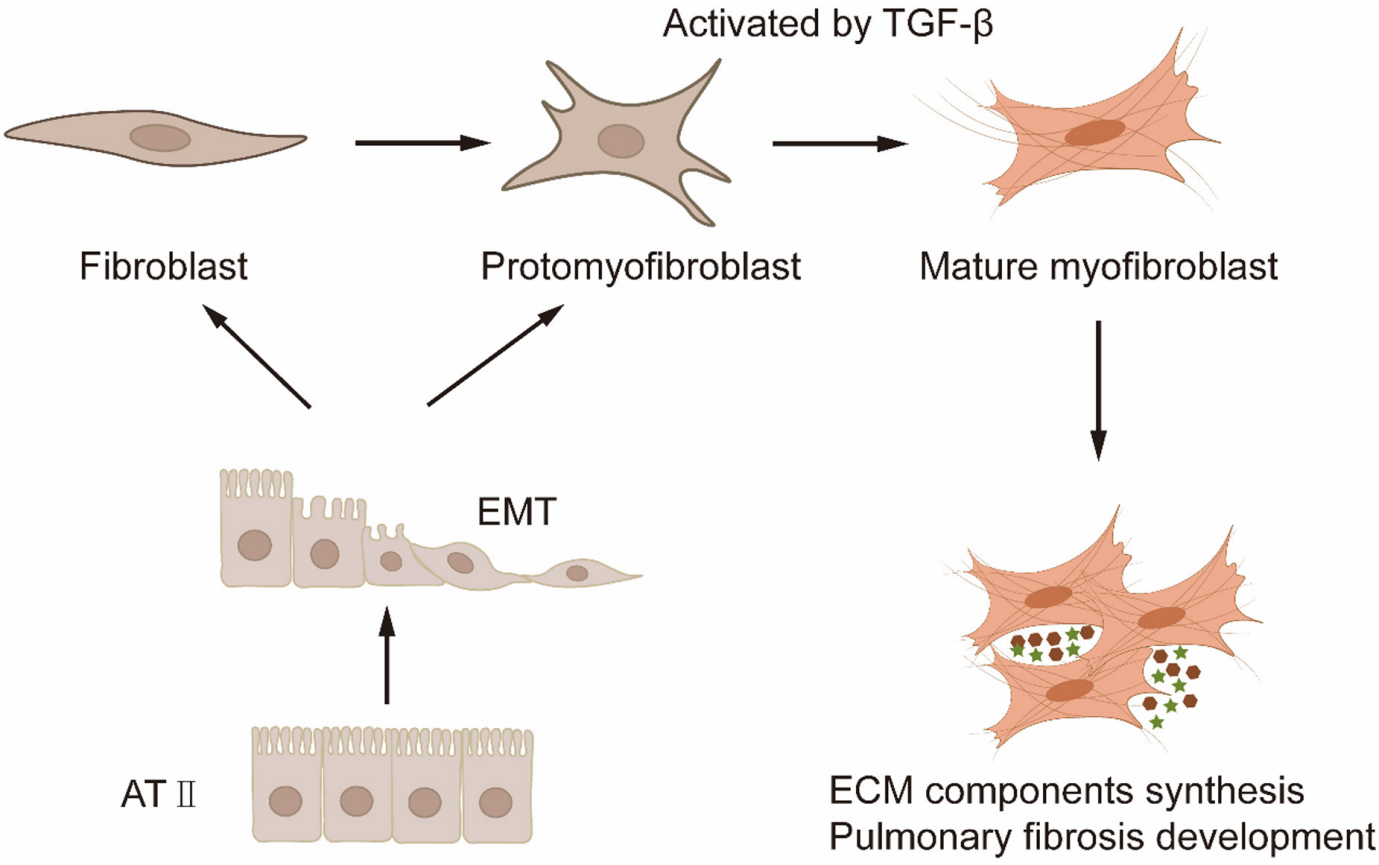
Overview of molecular mechanism of pulmonary fibrosis. Some alveolar type II cells may undergo the EMT process and differentiate into fibroblasts and myofibroblasts.^14^ Original and differentiated myofibroblasts are activated by TGF‐β and secrete ECM, leading to increased pulmonary fibrosis.

The hallmark of pulmonary fibrosis is the excessive deposition of collagen and ECM. The ECM of fibroblasts facilitates fibroblast activation and sustains pathology.^13^ The primary morphological features of pulmonary fibrosis result from an imbalance between two physiological processes in the lungs (Figure 1): (1) the proliferation/apoptosis of fibroblasts and myofibroblasts^14^; (2) the synthesis/degradation of ECM components.^15^ These processes are closely linked, with the disruption of fibroblasts' and myofibroblasts' normal functioning being a key driver of ECM homeostasis imbalance, thereby leading to the development of pulmonary fibrosis.^16^

The finding that alveolar epithelial cells (AECs) and fibroblasts in pulmonary fibrosis produce abnormal ECM implicates the TGF‐β signalling pathway.^17^ Aberrant regulation of the TGF‐β/SMAD pathway is identified as a significant pathogenic mechanism in pulmonary fibrosis.^18^, ^19^

Multiple integrins are implicated in pulmonary fibrosis: αvβ6 facilitates TGF‐β activation in AECs, while αvβ1 plays a similar role in myofibroblasts, which are essential in the development of fibrotic diseases.^20^, ^21^ Regardless of whether TGFβ, ECM, integrins or EMT influence pulmonary fibrosis, CTGF's involvement is evident. Thus, targeting CTGF has emerged as a novel direction for research in treating these conditions.

### 1.3 CTGF involved in pulmonary fibrosis

CTGF is a cysteine‐rich 38‐kDa peptide secreted from human umbilical vein endothelial cells (HUVECs) and belongs to the CCN family, also known as CCN2.^22^ It is a pleiotropic protein with significant fibrotic activity.^23^ TGF‐β, a key cytokine that promotes pulmonary fibrosis, works upstream of CTGF, which plays a vital role in the disease. Evidence of CTGF's contribution to pulmonary fibrosis includes (1) elevated levels of CCN2 in patients with pulmonary fibrosis and higher plasma CTGF levels compared to normal controls,^22^ (2) increased CTGF expression in mouse models of pulmonary fibrosis induced by bleomycin and radiation,^24^, ^25^ and (3) identification of CTGF as a critical gene in pulmonary fibrosis through meta‐analysis.^26^ This paper will detail the pathways through which CTGF influences pulmonary fibrosis.

#### 1.3.1 TGF‐β: a downstream protein of CTGF

TGF‐β plays a crucial role in the pathogenesis of pulmonary fibrosis by activating fibroblasts and stimulating ECM production.^27^ Alveolar macrophages are prompted to release substances such as CC chemokine ligand 2 (CCL2),^28^ oxidized phospholipids,^29^, ^30^ and citrullinated vimentin (Cit‐Vim),^31^ which enhance the expression of TGF‐β and CTGF, exacerbating pulmonary fibrosis. TGF‐β also promotes fibroblast proliferation, stimulates granulation tissue formation and collagen deposition.^32^

The multifunctional cytokine TGF‐β induces CTGF expression strongly through the Smad signalling pathway. CTGF is influenced by TGF‐β, resulting in a feedback loop amplifying TGF‐β levels in tissues.^33^ TGF‐β induced FN expression was decreased by CTGF siRNA transfection. TGF‐β initially influences extracellular signal‐regulated kinase (ERK) phosphorylation, followed by disintegrin and metalloproteinase 17 (ADAM17) phosphorylation. It also activates ribosomal S6 kinase 1 (RSK1), thus affecting the binding of enhancer‐binding protein β (C/EBPβ) to the CTGF promoter. This process ultimately regulates CTGF expression in human lung epithelial cells (A549).^34^ Recent research has demonstrated that CTGF is at the downstream of TGF‐β; still, it can affect the expression of TGF‐β. In a mouse model of fibrosis, there is a notable rise in CTGF expression when using an adenovirus vector encoding active TGF‐β (AbTGF‐β). The interaction between CTGF and TGF‐β necessitates a higher CTGF concentration to elevate fibrosis markers and TGF‐β expression individually, whereas a lower concentration achieves simultaneous induction of both TGF‐β and CTGF. This observation suggests a synergistic induction of pulmonary fibrosis by CTGF and TGF‐β.^33^ It has been reported that CTGF increases the activity of TGF‐β by binding to the N‐terminal structural domain of TGF‐β, resulting in a worsening of fibrosis.^33^ Some researchers posit that the interaction between TGF‐β ligands and receptors leads to Smad3 phosphorylation, forming a complex with Smad4. This complex binds the Smad‐binding element (SBE) in the CTGF proximal promoter, activating CTGF transcription.^6^

Inhibition of TGF‐β is a pivotal strategy in IPF treatment, as it can mitigate pulmonary fibrosis. For instance, current preclinical studies have shown that roxadustat administration reduces experimental pulmonary fibrosis by inhibiting TGF‐β1/Smad activation and decreasing CTGF expression.^35^

#### 1.3.2 Lipoprotein receptor (LRP): a receptor for CTGF

The low‐density LRP belongs to the low‐density lipoprotein (LDL) receptor family and has been identified as one of the receptors for CTGF. Immunoprecipitation data have shown that CTGF can bind to LRP, whereas cells deficient in the LRP gene are unable to bind to CTGF.^36^ CTGF is known to induce phosphorylation of lysine residues in LRP. Upon binding to LRP, CTGF not only triggers tyrosine phosphorylation in the asparagine‐proline‐any amino acid‐tyrosine (NPXY) motif within the cytoplasmic domain of LRP, initiating downstream signalling pathways with the assistance of specific linker proteins in the cytoplasm but also leads to the transport of CTGF to the intracellular lysosome through the cell membrane via a small concave region, where it is hydrolyzed, losing its biological activity. The expression of LRP has been found to correlate with CTGF in vitro experiments.^37^ In mouse hepatic stellate cells, LRP can activate CTGF, influencing cell adhesion and migration.^38^

LRP‐6 is co‐activated by CTGF and TGF‐β in renal fibrosis and influences fibrosis by enhancing the WNT/β‐catenin pathway.^39^ The Wnt/β‐catenin signalling pathway, found dysregulated in microarrays from patients with lung fibrosis, exacerbates pulmonary fibrosis when activated.^40^ It has been demonstrated that CTGF and TGF‐β synergistically enhance the expression of α‐SMA in fibroblasts in an LRP1‐dependent manner. The absence of LRP1 converts the antiproliferative effect of TGF‐β in fibroblasts into a proliferative effect.^41^ Betulinic acid (BA) significantly reduced the levels of Wnt3a and LRP6 in mice with bleomycin‐induced lung fibrosis.^42^

#### 1.3.3 Integrins: CTGF interaction affects fibrosis

Integrins, as transmembrane receptors, facilitate the connection between cells and their external environment.^43^ The integrin complex on the cell surface serves as the primary receptor for CTGF, and integrin‐linked kinase (ILK) plays a key role in mediating integrin signalling. CTGF binding to cell surface integrins activates ILK signalling.^44^ ILK has been identified as a regulator of epithelial‐mesenchymal transition (EMT) in various epithelia, including those of the kidney, ovary, lens and mammary glands.^45^, ^46^ Overexpression of CTGF in AT II cells has been shown to increase ILK gene and protein levels and silencing ILK with siRNA significantly reduced both CTGF and fibronectin levels, suggesting that EMT can mediate the effects of CTGF in cells via ILK.^47^ Typically, lung fibroblasts differentiate from other cell types; however, periostin signalling can induce myofibroblast differentiation by prompting fibroblasts to release pro‐fibrotic mediator CTGF via beta‐1 integrin.^48^, ^49^ CCN2 enhances fibronectin adhesion through integrin α5β1.^50^ Pre‐adipocyte factor‐1 (Pref‐1) significantly influences airway fibrosis in patients with chronic obstructive asthma through the integrin receptor 51/ERK/AP‐1 signalling pathway, inducing CTGF expression in human lung fibroblasts.^51^ CTGF increases chondrosarcoma cell migration by enhancing MMP‐13 expression through αVβ1 integrin. CTGF disrupts alveolarization and induces pulmonary fibrosis through β3 integrin, FAK, ERK and NF‐κB signalling pathways.^52^ In murine models of liver fibrosis, MMP‐13 has been shown to promote fibrosis,^53^ whereas it can reduce overall ECM deposition in IPF.^54^ The specific mechanism by which CTGF influences MMP‐13 in pulmonary fibrosis requires further investigation. CTGF also interacts with other integrins to influence fibrosis in various organs, such as αvβ1 in liver and kidney fibrosis,^55^, ^56^, ^57^ α5β1 in the adhesion and migration of activated pancreatic stellate cells,^58^ and αvβ1 in gingival fibrosis.^59^ Although these interactions have not been demonstrated in pulmonary fibrosis, they provide a direction for future research.

Inhibitors targeting αvβ1 have been shown to mitigate bleomycin‐induced lung fibrosis and carbon tetrachloride‐induced liver fibrosis.^43^ The αvβ6 integrin is a crucial in vivo activator of TGF‐β in the lung, and inhibition of αvβ6 has been found to improve pulmonary fibrosis.^60^

#### 1.3.4 ECM: CTGF's influence

A notable characteristic of pulmonary fibrosis is the improper deposition of ECM.^61^ The ECM is a three‐dimensional, non‐cellular complex structure present in all tissues and vital for life. The remodelling of the ECM, mediated by CTGF, hinders muscle regeneration.^62^ A wide range of ECM proteins exists, most of which are linked to pulmonary fibrosis. Studies have shown that suppressing CTGF expression in lung fibroblasts significantly reduces fibronectin and type 1 collagen significantly.^63^ In models of pulmonary fibrosis induced by bleomycin, mice with a knockout of CTGF exhibit less collagen deposition than their wild‐type counterparts.^63^, ^64^ CTGF is known to prompt collagen expression via the JNK pathway. The treatment of lung fibroblasts with CTGF activates the Rac1/MLK3/JNK signalling pathway, leading to the activation of AP‐1 and the recruitment of c‐Jun and c‐Fos to the promoter of collagen I, ultimately stimulating the expression of collagen I in human lung fibroblasts.^65^ Furthermore, the downregulation of miR‐26a, resulting in the post‐transcriptional repression of CTGF, has been reported to encourage the differentiation of MRC‐5 human fetal lung fibroblasts into pathological myofibroblasts and to promote collagen production.^66^ The relationship between CTGF and ECM proteins, capable of positive or negative feedback, is highly intricate. The detailed mechanisms underlying these interactions remain to be further elucidated.

#### 1.3.5 EMT: facilitated by CTGF

EMT, a process through which cells lose their epithelial characteristics and gain mesenchymal traits, is pivotal in mammalian growth and development, wound healing and cancer metastasis.^67^, ^68^ During EMT, the expression of mesenchymal markers such as α‐SMA, fibroblast‐specific protein‐1 (FSP1), vimentin and desmin increases, indicating increased pulmonary fibrosis.^69^, ^70^, ^71^ In vitro studies have demonstrated that mediators like TGF‐β and CTGF can induce EMT in human epithelial cells, thereby facilitating fibrogenesis via ECM production.^72^ Treatment with TGF‐β leads to reduced E‐cadherin levels and increased expression of specific mesenchymal markers. Suppression of CTGF through siRNA‐mediated approaches reduces the expression of α‐SMA and restores E‐cadherin levels.^73^ Citrulline vimentin has been found to stimulate CTGF expression and increase its levels in primary lung fibroblasts.^31^ The suppression of CTGF expression in human embryonic fibroblasts has been shown to inhibit α‐SMA and vimentin expression.^74^ In mice genetically modified to overexpress CTGF, an overexpression of mesenchymal cell markers was observed, suggesting that EMT occurred.^73^, ^75^ Subsequent experiments have indicated that CTGF encourages EMT through the ILK pathway.^47^ miR‐30c‐5p, a microRNA that usually ranges from 18 to 24 nucleotides in length and targets CTGF, has been shown to hinder the EMT process in A549 cells by influencing CTGF and ATG5‐related autophagy.^76^ CTGF is instrumental in EMT and affects the progression of pulmonary fibrosis.

### 1.4 Drugs targeting CTGF for pulmonary fibrosis

**FIGURE 2:**
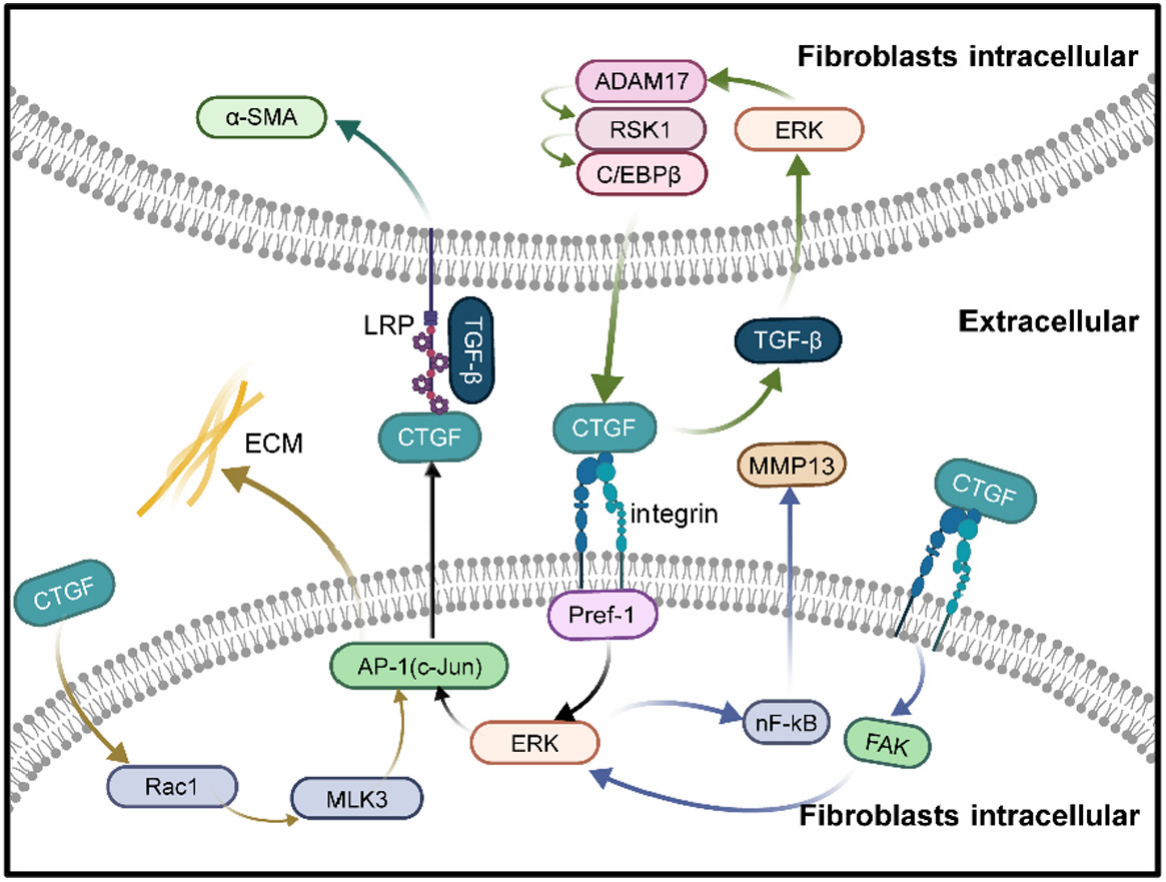
Schematic summary of the mechanisms involved in CTGF in pulmonary fibrosis. CTGF is secreted from fibroblasts into the extracellular space and acts on fibroblasts. (1) CTGF/RAC1/MLK3/AP‐1 affects the components of ECM and aggravates pulmonary fibrosis; (2) Differentiation of lung fibroblasts is induced by CTGF/Pref‐1/α5β1/ERK/AP‐1; (3) TGF‐β induces CTGF expression through ERK/ADAM17/RSK1/ C/EBP‐β pathway; (4) CTGF promotes upregulation of MMP‐13 through αvβ3 /FAK/ERK/NF‐κB dependent pathway and aggravates pulmonary fibrosis.

CTGF is significantly involved in pulmonary fibrosis (Figure 2) and fibrosis in other organs, presenting a potential target for treatment. Several drugs aimed at CTGF are currently under investigation. Research on fibrosis has utilized many CTGF‐targeting molecules, including antibodies, siRNA, short hairpin RNA (shRNA) and natural compounds (Table 1).^8^, ^77^ A nanotherapeutic approach using CTGF siRNA‐DegradaBALL (LEM‐S401) has been demonstrated to inhibit CTGF/CCN‐2 effectively and persistently for treating skin fibrosis.^78^ Anti‐CTGF oligonucleotides have been applied in treating hyperplastic keloid scars.^79^ Pamrevlumab, a recombinant human antibody against CTGF, has shown promise as a treatment for IPF and has entered phase 3 clinical trials, making it one of the most noted agents.^80^, ^81^ The use of Anti‐CTGF single‐chain variable fragment antibody (anti‐CTGF scFv) has been reported to significantly lessen the severity of pulmonary fibrosis in mice, highlighting its therapeutic potential.^82^ There might be additional therapeutic agents for treating pulmonary fibrosis through the CTGF pathway. This section reviews these potential drugs (Table 2). Atorvastatin, typically used as a hypolipidemic agent, was found in one study to mitigate lung fibrosis by lowering CTGF expression in a mouse model of bleomycin‐induced lung fibrosis.^83^

**TABLE 1:** Drugs that target CTGF.

Drug name	Description	Stage	References
Atorvastatin	Inhibition of CTGF (CCN2)/ERK signalling pathway	Preclinical	83, 84
Pamrevlumab	Peptides targeting CTGF	Phase III	80, 85
siRNA‐DegradaBALL (LEM‐S401)	CTGF‐Targeting siRNA can treat skin fibrosis	Preclinical	^78^
BLR‐100/BLR‐200	CTGF‐Targeting Peptides can treat PDAC	Preclinical	^86^
RXI‐109	CTGF‐Targeting siRNA can treat subretinal fibrosis	PhaseI/II	^87^

**TABLE 2:** Pulmonary fibrosis drugs target CTGF‐related pathways.

Drug name	Description	References
Roxadustat	Inhibition of the TGF‐β1/Smad pathway decreased CTGF	^35^
Thalidomide	Inhibition of TGF‐β1 induced ECM	^88^
Adiponectin	Reduces paraquat‐induced TGF‐β1 and α‐SMA	^89^
Nagilactone D	Improving lung fibrosis by regulating the TGF‐β/Smad signalling pathway	^90^
Gentiopicroside (GPS)	Reduction of TGF‐β1 and CTGF expression in mice with pulmonary fibrosis	^91^
Withaferin A	Inhibition of CTGF expression, and TGF‐β and Smad phosphorylation	^92^
Pioglitazone	Inhibits the expression of TNF‐α, procollagen I and CTGF	^93^
Nervilia fordii Extract	Inhibition of TGF‐β/Smad signalling pathway	^94^

## 2 CONCLUSION

Pulmonary fibrosis remains a critical health issue that necessitates immediate attention. Factors such as the environment, age and genetics can influence the progression of pulmonary fibrosis, a condition characterized by a high mortality rate and a median survival time of 3–5 years post‐diagnosis. Since the outbreak in 2019, COVID‐19 has significantly affected daily life. In severe instances, COVID‐19 impairs the respiratory system, leading to pneumonia and pulmonary fibrosis. Research has indicated that COVID‐19 exacerbates fibrosis in individuals with pulmonary fibrosis,^95^ and elevated levels of CTGF and TGF‐β have been observed in AECs infected with SARS‐CoV‐2.^96^ Anti‐CTGF therapy, as a potential treatment for fibrosis, is expected to mitigate pulmonary fibrosis in severe COVID‐19 cases and aid in recovery.^77^ CTGF is crucial in the development of pulmonary fibrosis.

TGF‐β is recognized for its vital role in the progression of pulmonary fibrosis, influencing the disease through various mechanisms. As a protein downstream of TGF‐β, CTGF is significant in pulmonary fibrosis across multiple pathways. There is a positive feedback loop between CTGF and TGF‐β that intensifies fibrosis. Current investigations into drugs have revealed that CTGF antibodies can curtail pulmonary fibrosis via the TGF‐β pathway, with these drugs now in phase 3 clinical trials. Additionally, CTGF can interact with integrins, affecting the emergence of EMT and the accumulation of the ECM; thus, influencing pulmonary fibrosis. Various potential miRNAs or drugs targeting CTGF impact integrins and EMT. LRP and CTGF play a specific role in the fibrosis of other organs together. Yet, the function of CTGF and LRP in pulmonary fibrosis remains unconfirmed, presenting an interesting avenue for future research. While many mechanisms are still to be clarified, compiling potential drugs targeting CTGF provides insights into the development of treatments for pulmonary fibrosis and offers a valuable perspective for future studies on the mechanisms driving the progression of the disease.

## AUTHOR CONTRIBUTIONS

**Yudan Qiu:** Writing – original draft (lead); writing – review and editing (lead). **Yueyue Que:** Writing – review and editing (equal). **Zheyu Ding:** Software (equal). **Shanshan Zhang:** Visualization (equal). **Rong Wei:** Resources (equal). **Jianing Xia:** Resources (equal). **Yingying Lin:** Data curation (lead); funding acquisition (lead); supervision (lead).

## FUNDING INFORMATION

This study supported by The National Natural Science Foundation of China (32100620).

## CONFLICT OF INTEREST STATEMENT

The authors confirm that there are no conflicts of interest.

## Data Availability

Data sharing not applicable to this article as no datasets were generated or analysed during the current study.
